# Notes and News

**Published:** 1900-02-17

**Authors:** 


### Notes and News.

### HOSPITAL MEETINGS.

London Fever Hospital.—Annual Meeting at five p m., on Friday, February 16th, at the Hotel Victoria.

Charing Cross Hospital.—Annual General Court at two p.m., on Wednesday, February 21st, in the Board Room of the Hospital.

Middlesex Hospital. — Annual Meeting at twelve noon, on Thursday, February 22nd, at the Hospital.

Central London Ophthalmic Hospital.—Annual Meeting on Thursday, February 22nd, at the Hospital.

King's College Hospital. — Annual Court at four p.m., on Thursday, February 22nd, at the Hospital.

Metropolitan Hospital.—Anniversary Festival Dinner at seven p.m , on Thursday, February 22nd, at the Whitehall Rooms, Hotel Metropole.

City of London Lying-in Hospital —Annual Meeting at three p.m., on Wednesday, February 21st, at the Hospital.

The Longmore Hospital for Incurables has received a gift of £2,500 from Mr. Thomas Aitkin, of Leith. The institution serves Edinburgh and the whole of the South-East of Scotland.

Chatelier Eugene, J.A., M.B., C.M.Edin., has been appointed medical superintendent of the City Hospital, Birmingham, *vice* C. K. Millard, M.D., D.S.C., resigned.

The London and South-Western Railway Company are providing "Red Cross" trains to convey the sick and wounded to Netley and Haslar Hospitals. The first one completed contained 12 stretcher beds, and seating room for 18 patients besides.

The London hospitals will be the richer by upwards of £80,000 through the will of Mr. Siegfried Zunz, of Wimbledon. The choice of the institutions to benefit is left to the discretion of the executors, who are directed to found wards to the memory of Mr. Zunz's wife. The amount of money at first available will be £25,000, to be devoted to one institution for the purpose mentioned.

The site of the Yeomanry Hospital is to be near De Aar. This will bring a base hospital considerably nearer the actual operations of the war than has yet been the case. The arrangements for a Yeomanry Field Hospital are also rapidly advancing. The plan of encouraging the endowment of individual beds in the hospital is succeeding excellently. Mr. James Elliman, of Slough, has given 10 beds, and promises to maintain them if necessary for 12 months.

Dr. William Pasteur, F.R.C.P., M.R.C.S., Assistant Physician to the Middlesex Hospital, and Dean of the School, has been elected Physician to the Hospital.

The memorial-stone of a new Lady Margaret Hospital for Infectious Diseases has been laid at Millport, Scotland, by Sir Charles Dalrymple.

The influence of the war is so universal that warlike phraseology and methods find their way into charitable institutions. In a Welsh paper there is an amusing account of how a special meeting of a district council was convened. At a meeting being held business concerning a hospital was brought forward, and it was found that the presence of the majority of the council was necessary, which was not the case. Accordingly, we learn from the Press that the various members present started out on a scouting party to commandeer their truant comrades, and that after an hour's hunt the scouts returned with their prize.

It was almost a foregone conclusion that Sir Michael Foster would be elected Member of Parliament for the London University. He secured 1,271 votes, Dr. Collins coming next with 863. The distinguished career of Sir Michael Foster renders him eminently suitable to represent the University. His work has been well known since 1860, in which year he became Professor of Practical Physiology at University College, and Fullerian Professor of Physiology at the Royal Institute. In 1870 he was elected Fellow of and Prelector at Trinity College, Cambridge, and in 1883 he became Professor of Physiology at Cambridge.

How serious a matter influenza may become when it breaks out in an institution the inmates of which are of poor resisting power, and the arrangements of which are such as to put isolation out of the question, is shown by the record of the deaths in the Leavesden Asylum during the past six week. At the meeting of the Metropolitan Asylums Board last Saturday attention was drawn to the largely increased death-rate among the inmates at Leavesden Asylum, 96 deaths having occurred in six weeks; and, in explanation, it was stated by the chairman of the committee that there had been an outbreak of influenza in that institution, and that 49 of the deaths mentioned were due to that disease.

Another instance of the probable influence of contaminated shell-fish in producing typhoid fever is reported from Belfast. That city has long had more than its share of typhoid fever, which a couple of years ago rose into quite an epidemic, and it is suggested that the consumption of cockles, gathered as they are on the shores of estuaries, contaminated by sewage, may be the cause of this continued prevalence of fever. As confirming this idea, it is stated that although the fever prevails in more than their due proportion among the poorer classes, the Jews, who are mostly poor, have escaped the disease. There are, says a writer in the *Lancet*, about 700 Jews in Belfast, mostly of Russian origin, and poor, who all strictly adhere to the Mosaic code of diet, discarding amongst other food all animals living in the sea and not covered by scales, and it seems that no case of typhoid fever has been reported amongst the members of the congregation for many years past.

